# Supplementary material for: A Likelihood Approach for Real-Time Calibration of Stochastic Compartmental Epidemic Models
Source: PLoS Comput Biol. 2017 Jan 17;13(1):e1005257. doi: 10.1371/journal.pcbi.1005257 (PMC5240920; doi:10.1371/journal.pcbi.1005257)
Supplement: S1 File — (TAR.GZ) [file pcbi.1005257.s014.tar.gz › HSPH_Online-SI-Revision/EnKF07/n1-mild/n1-mild_EnKF2_table-4.pdf]

1	newly 3 weeks spec	24.	18.34	{0., 28.}	116.417	0.983099	newly 3 weeks cumul	78.	107.3	{13., 334.}	133.385	0.529883	newly 5 weeks spec	32.	5.1	{0., 14.}	91.375	1.6466	newly 5 weeks cumul	149.	122.83	{13., 392.}	107.55	0.686793	newly 59 weeks cumul	331.	128.81	{13., 460.}	89.2296	0.925616
2	newly 3 weeks spec	34.	14.35	{0., 19.}	104.265	1.32177	newly 3 weeks cumul	90.	92.35	{6., 133.}	126.767	0.697929	newly 5 weeks spec	17.	4.25	{0., 8.}	95.	1.6177	newly 5 weeks cumul	145.	104.93	{7., 136.}	111.814	0.824613	newly 55 weeks cumul	202.	112.77	{7., 310.}	103.243	0.916132
3	newly 3 weeks spec	20.	55.03	{7., 116.}	201.25	0.517098	newly 3 weeks cumul	57.	356.32	{157., 856.}	528.351	0.728846	newly 5 weeks spec	25.	16.47	{0., 39.}	67.64	0.750099	newly 5 weeks cumul	111.	403.35	{169., 934.}	266.982	0.508579	newly 64 weeks cumul	238.	426.32	{181., 971.}	91.2185	0.257962
4	newly 3 weeks spec	15.	13.86	{0., 23.}	118.533	1.04019	newly 3 weeks cumul	42.	93.61	{9., 133.}	166.405	0.341684	newly 5 weeks spec	9.	5.97	{0., 9.}	114.778	1.24769	newly 5 weeks cumul	71.	108.84	{11., 151.}	123.972	0.387743	newly 58 weeks cumul	398.	122.67	{11., 179.}	87.5251	0.932139
5	newly 3 weeks spec	50.	20.35	{0., 44.}	99.7	1.37256	newly 3 weeks cumul	122.	141.09	{19., 389.}	111.779	0.493842	newly 5 weeks spec	37.	7.16	{0., 10.}	92.2703	1.76467	newly 5 weeks cumul	231.	160.41	{21., 480.}	94.645	0.659055	newly 60 weeks cumul	369.	173.42	{21., 539.}	86.7425	0.793491
6	newly 3 weeks spec	26.	55.74	{5., 126.}	156.385	0.543134	newly 3 weeks cumul	47.	374.18	{148., 814.}	698.723	0.816651	newly 5 weeks spec	37.	18.26	{0., 40.}	68.4865	0.901917	newly 5 weeks cumul	121.	424.09	{165., 914.}	255.314	0.48806	newly 64 weeks cumul	400.	449.83	{166., 978.}	52.0975	0.230787
7	newly 3 weeks spec	25.	41.36	{3., 71.}	116.48	0.496533	newly 3 weeks cumul	44.	288.85	{95., 750.}	560.114	0.715441	newly 5 weeks spec	48.	12.31	{0., 34.}	79.3958	1.15794	newly 5 weeks cumul	142.	323.49	{104., 875.}	138.613	0.312238	newly 63 weeks cumul	381.	343.47	{124., 918.}	60.2546	0.315311
8	newly 3 weeks spec	13.	4.28	{0., 9.}	86.7692	1.18821	newly 3 weeks cumul	44.	56.07	{6., 66.}	112.659	0.429942	newly 5 weeks spec	14.	1.63	{0., 4.}	93.0714	1.74145	newly 5 weeks cumul	78.	60.	{6., 83.}	95.9487	0.591224	newly 55 weeks cumul	449.	64.41	{6., 125.}	88.9198	1.23528
9	newly 3 weeks spec	32.	20.8	{0., 87.}	111.	1.17493	newly 3 weeks cumul	72.	121.53	{5., 325.}	161.875	0.549595	newly 5 weeks spec	42.	8.77	{0., 24.}	91.119	1.66154	newly 5 weeks cumul	158.	143.45	{8., 630.}	116.12	0.723695	newly 54 weeks cumul	322.	158.65	{8., 657.}	92.4255	0.906535
10	newly 3 weeks spec	37.	8.49	{0., 21.}	89.2703	1.53046	newly 3 weeks cumul	113.	91.25	{13., 305.}	98.7345	0.582807	newly 5 weeks spec	27.	2.78	{0., 6.}	94.7407	1.85317	newly 5 weeks cumul	184.	98.42	{13., 337.}	89.0109	0.713179	newly 55 weeks cumul	266.	105.21	{13., 337.}	85.1466	0.819332
11	newly 3 weeks spec	20.	13.39	{0., 25.}	122.35	1.25336	newly 3 weeks cumul	44.	96.16	{3., 297.}	204.318	0.534659	newly 5 weeks spec	18.	5.18	{0., 7.}	102.444	1.61907	newly 5 weeks cumul	86.	109.96	{4., 340.}	145.023	0.681642	newly 54 weeks cumul	455.	116.2	{4., 340.}	88.7516	1.18474
12	newly 3 weeks spec	14.	8.07	{0., 15.}	93.7857	1.04363	newly 3 weeks cumul	27.	73.15	{9., 116.}	204.407	0.340537	newly 5 weeks spec	18.	2.91	{0., 6.}	92.0556	1.53272	newly 5 weeks cumul	68.	80.49	{10., 124.}	108.397	0.441478	newly 55 weeks cumul	321.	84.86	{10., 143.}	86.1682	0.945963
13	newly 3 weeks spec	16.	15.85	{0., 19.}	132.813	0.99606	newly 3 weeks cumul	60.	105.03	{11., 276.}	154.817	0.482623	newly 5 weeks spec	15.	4.6	{0., 6.}	103.067	1.53921	newly 5 weeks cumul	111.	117.85	{11., 319.}	118.568	0.621659	newly 56 weeks cumul	193.	124.53	{11., 335.}	100.44	0.782925
14	newly 3 weeks spec	29.	28.05	{0., 97.}	131.345	1.14015	newly 3 weeks cumul	93.	184.55	{12., 682.}	172.591	0.479306	newly 5 weeks spec	30.	10.76	{0., 34.}	95.9333	1.59086	newly 5 weeks cumul	159.	213.67	{13., 843.}	133.491	0.576348	newly 60 weeks cumul	260.	235.51	{14., 893.}	106.965	0.683906
15	newly 3 weeks spec	25.	24.42	{0., 88.}	130.72	1.10404	newly 3 weeks cumul	71.	175.23	{16., 679.}	200.662	0.418175	newly 5 weeks spec	20.	9.52	{0., 29.}	108.8	1.49601	newly 5 weeks cumul	129.	199.18	{16., 773.}	141.473	0.51368	newly 60 weeks cumul	389.	217.89	{19., 884.}	89.0977	0.782852
16	newly 3 weeks spec	23.	8.35	{0., 13.}	98.3043	1.42868	newly 3 weeks cumul	52.	102.12	{6., 125.}	164.308	0.474016	newly 5 weeks spec	7.	2.08	{0., 4.}	98.8571	1.39155	newly 5 weeks cumul	83.	108.63	{6., 136.}	126.036	0.563751	newly 58 weeks cumul	349.	111.75	{7., 143.}	90.5587	1.03066
17	newly 3 weeks spec	17.	10.06	{0., 20.}	84.3529	0.929596	newly 3 weeks cumul	43.	144.84	{15., 386.}	265.256	0.496909	newly 5 weeks spec	15.	2.2	{0., 4.}	94.9333	1.72718	newly 5 weeks cumul	78.	151.27	{16., 398.}	136.346	0.403497	newly 61 weeks cumul	332.	153.8	{16., 401.}	71.5301	0.647339
18	newly 3 weeks spec	18.	5.56	{0., 9.}	93.	1.21929	newly 3 weeks cumul	52.	65.04	{6., 72.}	126.077	0.497862	newly 5 weeks spec	18.	1.82	{0., 4.}	94.8889	1.74333	newly 5 weeks cumul	99.	69.72	{6., 117.}	103.273	0.683234	newly 54 weeks cumul	320.	77.92	{6., 123.}	90.6375	1.08967
19	newly 3 weeks spec	40.	8.63	{0., 24.}	80.575	1.17888	newly 3 weeks cumul	125.	142.85	{19., 391.}	82.52	0.385451	newly 5 weeks spec	35.	1.75	{0., 5.}	95.	1.99771	newly 5 weeks cumul	206.	148.29	{19., 398.}	71.1796	0.488721	newly 61 weeks cumul	389.	150.66	{20., 400.}	70.0154	0.67282
20	newly 3 weeks spec	37.	15.24	{0., 30.}	102.757	1.56278	newly 3 weeks cumul	71.	117.63	{6., 403.}	158.38	0.533047	newly 5 weeks spec	33.	4.79	{0., 8.}	97.4242	1.993	newly 5 weeks cumul	150.	130.81	{6., 405.}	117.287	0.742895	newly 58 weeks cumul	270.	138.98	{6., 405.}	99.9852	0.922927
21	newly 3 weeks spec	20.	6.46	{0., 11.}	92.5	1.4572	newly 3 weeks cumul	49.	131.78	{10., 318.}	211.184	0.402431	newly 5 weeks spec	20.	0.86	{0., 3.}	95.7	1.94529	newly 5 weeks cumul	108.	134.7	{10., 318.}	118.241	0.51826	newly 58 weeks cumul	233.	135.78	{10., 318.}	91.03	0.740574
22	newly 3 weeks spec	7.	18.18	{0., 39.}	246.286	0.795957	newly 3 weeks cumul	28.	142.86	{15., 329.}	425.429	0.475629	newly 5 weeks spec	13.	6.99	{0., 15.}	114.692	1.42705	newly 5 weeks cumul	64.	160.59	{15., 470.}	191.891	0.351833	newly 60 weeks cumul	439.	174.76	{15., 557.}	85.0934	0.836729
23	newly 3 weeks spec	19.	13.6	{0., 16.}	121.053	1.05638	newly 3 weeks cumul	44.	77.24	{5., 96.}	164.955	0.500513	newly 5 weeks spec	23.	5.2	{0., 7.}	98.	1.68056	newly 5 weeks cumul	99.	90.52	{6., 154.}	121.535	0.715895	newly 55 weeks cumul	317.	102.25	{6., 254.}	92.653	1.08204
24	newly 3 weeks spec	25.	7.5	{0., 12.}	87.84	1.22448	newly 3 weeks cumul	65.	81.39	{7., 82.}	121.8	0.496678	newly 5 weeks spec	28.	3.65	{0., 8.}	90.6786	1.64787	newly 5 weeks cumul	129.	89.95	{7., 124.}	99.4186	0.681984	newly 57 weeks cumul	399.	103.09	{7., 154.}	89.2356	1.04251
25	newly 3 weeks spec	15.	12.69	{0., 12.}	116.467	0.792395	newly 3 weeks cumul	35.	65.92	{15., 89.}	147.543	0.305814	newly 5 weeks spec	9.	6.93	{0., 9.}	122.556	1.13976	newly 5 weeks cumul	63.	82.07	{17., 127.}	126.587	0.441253	newly 54 weeks cumul	293.	96.47	{19., 128.}	93.2184	0.946069
26	newly 3 weeks spec	28.	9.31	{0., 14.}	93.5357	1.34039	newly 3 weeks cumul	64.	84.45	{3., 173.}	131.953	0.601787	newly 5 weeks spec	24.	5.45	{0., 10.}	96.375	1.65099	newly 5 weeks cumul	119.	97.24	{4., 215.}	107.664	0.754887	newly 57 weeks cumul	217.	109.67	{4., 215.}	94.0138	0.910844
27	newly 3 weeks spec	24.	8.96	{0., 10.}	107.917	1.35982	newly 3 weeks cumul	69.	53.34	{10., 46.}	106.58	0.603814	newly 5 weeks spec	20.	2.08	{0., 4.}	95.7	1.84178	newly 5 weeks cumul	118.	59.87	{10., 50.}	100.178	0.772036	newly 54 weeks cumul	267.	64.91	{10., 58.}	91.9738	1.04931
28	newly 3 weeks spec	11.	18.14	{0., 46.}	176.	0.966879	newly 3 weeks cumul	24.	156.	{22., 375.}	561.	0.561049	newly 5 weeks spec	16.	5.62	{0., 16.}	100.375	1.58639	newly 5 weeks cumul	66.	171.56	{25., 500.}	200.697	0.374495	newly 60 weeks cumul	343.	181.76	{26., 577.}	87.9242	0.751701
29	newly 3 weeks spec	36.	11.32	{0., 11.}	100.111	1.34303	newly 3 weeks cumul	105.	89.57	{5., 195.}	117.038	0.717869	newly 5 weeks spec	22.	5.45	{0., 8.}	95.7727	1.54585	newly 5 weeks cumul	167.	102.72	{5., 195.}	103.82	0.808116	newly 57 weeks cumul	288.	116.98	{5., 195.}	92.7292	0.928071
30	newly 3 weeks spec	28.	12.62	{0., 25.}	84.2143	1.08858	newly 3 weeks cumul	83.	132.81	{13., 286.}	104.253	0.353076	newly 5 weeks spec	27.	3.72	{0., 7.}	91.7037	1.74765	newly 5 weeks cumul	148.	143.32	{13., 342.}	73.3919	0.402228	newly 61 weeks cumul	335.	148.66	{13., 375.}	72.8776	0.647064
31	newly 3 weeks spec	56.	15.62	{0., 35.}	89.1786	1.27878	newly 3 weeks cumul	151.	105.67	{6., 235.}	102.086	0.745864	newly 5 weeks spec	34.	5.01	{0., 15.}	92.5	1.70316	newly 5 weeks cumul	244.	120.25	{9., 376.}	93.7336	0.864483	newly 59 weeks cumul	353.	130.17	{9., 406.}	88.8017	0.964716
32	newly 3 weeks spec	11.	7.63	{0., 15.}	82.2727	0.794892	newly 3 weeks cumul	27.	76.32	{4., 67.}	226.074	0.366748	newly 5 weeks spec	24.	5.63	{0., 11.}	89.5417	1.39345	newly 5 weeks cumul	76.	87.91	{6., 86.}	116.539	0.513908	newly 57 weeks cumul	391.	113.89	{6., 203.}	87.9616	1.00009
33	newly 3 weeks spec	37.	8.96	{0., 15.}	97.2973	1.7303	newly 3 weeks cumul	84.	98.34	{8., 115.}	122.762	0.534567	newly 5 weeks spec	32.	2.59	{0., 6.}	96.4688	2.06381	newly 5 weeks cumul	159.	105.09	{8., 128.}	103.252	0.732925	newly 58 weeks cumul	332.	108.47	{8., 131.}	93.3404	0.992002
34	newly 3 weeks spec	13.	13.59	{0., 26.}	128.538	0.999467	newly 3 weeks cumul	35.	118.42	{2., 355.}	298.114	0.50637	newly 5 weeks spec	9.	5.78	{0., 9.}	113.556	1.2326	newly 5 weeks cumul	63.	132.51	{2., 400.}	198.556	0.579344	newly 57 weeks cumul	230.	151.39	{5., 611.}	110.291	0.927339
35	newly 3 weeks spec	24.	17.69	{0., 31.}	111.208	1.01276	newly 3 weeks cumul	77.	104.29	{7., 254.}	127.675	0.535267	newly 5 weeks spec	30.	6.07	{0., 12.}	94.0333	1.68172	newly 5 weeks cumul	154.	120.49	{7., 399.}	103.344	0.713971	newly 59 weeks cumul	405.	128.69	{7., 466.}	86.6691	1.01051
36	newly 3 weeks spec	51.	11.05	{0., 21.}	84.5294	1.31686	newly 3 weeks cumul	141.	137.35	{17., 346.}	78.078	0.430208	newly 5 weeks spec	46.	2.83	{0., 6.}	94.3261	2.08473	newly 5 weeks cumul	242.	145.96	{17., 364.}	73.6694	0.574785	newly 61 weeks cumul	339.	150.52	{17., 382.}	72.5133	0.671786
37	newly 3 weeks spec	18.	11.22	{0., 15.}	104.444	1.17904	newly 3 weeks cumul	52.	116.22	{4., 182.}	201.962	0.492873	newly 5 weeks spec	33.	4.89	{0., 10.}	93.7273	1.78393	newly 5 weeks cumul	122.	128.	{5., 284.}	129.82	0.708936	newly 57 weeks cumul	268.	140.58	{5., 287.}	100.127	0.907611
38	newly 3 weeks spec	43.	11.31	{0., 24.}	84.2093	1.23037	newly 3 weeks cumul	95.	130.99	{18., 329.}	88.8316	0.342126	newly 5 weeks spec	60.	2.99	{0., 6.}	95.0167	2.13081	newly 5 weeks cumul	220.	140.51	{18., 343.}	70.3682	0.512873	newly 61 weeks cumul	371.	146.2	{18., 343.}	72.3342	0.673148
39	newly 3 weeks spec	35.	16.09	{0., 36.}	92.9429	1.02985	newly 3 weeks cumul	95.	119.21	{9., 193.}	123.316	0.560541	newly 5 weeks spec	16.	5.19	{0., 10.}	93.6875	1.38217	newly 5 weeks cumul	151.	133.06	{11., 273.}	105.868	0.657748	newly 59 weeks cumul	278.	145.91	{13., 335.}	92.4856	0.824816
40	newly 3 weeks spec	40.	27.21	{1., 51.}	82.025	0.697262	newly 3 weeks cumul	91.	203.89	{32., 438.}	153.835	0.346216	newly 5 weeks spec	51.	8.73	{0., 20.}	88.4902	1.55841	newly 5 weeks cumul	193.	227.35	{35., 465.}	86.1813	0.365292	newly 62 weeks cumul	329.	254.47	{36., 511.}	75.5167	0.476243
41	newly 3 weeks spec	26.	38.24	{0., 77.}	110.769	0.573298	newly 3 weeks cumul	100.	287.61	{93., 655.}	196.03	0.390183	newly 5 weeks spec	38.	13.67	{0., 42.}	78.5	1.14401	newly 5 weeks cumul	177.	322.52	{110., 702.}	105.22	0.287436	newly 63 weeks cumul	284.	348.52	{115., 819.}	69.9085	0.280614
42	newly 3 weeks spec	58.	34.43	{2., 93.}	85.1897	0.772787	newly 3 weeks cumul	126.	236.67	{35., 626.}	130.77	0.368031	newly 5 weeks spec	44.	11.38	{0., 33.}	86.2273	1.42197	newly 5 weeks cumul	229.	267.39	{36., 712.}	91.0437	0.422534	newly 62 weeks cumul	291.	295.28	{38., 794.}	85.0722	0.462908
43	newly 3 weeks spec	61.	46.82	{2., 99.}	61.9344	0.550988	newly 3 weeks cumul	155.	413.19	{125., 1019.}	176.329	0.383492	newly 5 weeks spec	49.	13.2	{0., 38.}	76.	1.23727	newly 5 weeks cumul	274.	451.24	{149., 1057.}	89.4015	0.281774	newly 64 weeks cumul	392.	470.58	{165., 1098.}	59.9235	0.257585
44	newly 3 weeks spec	34.	11.99	{0., 14.}	99.5	1.3509	newly 3 weeks cumul	64.	87.91	{7., 174.}	145.109	0.572691	newly 5 weeks spec	35.	4.35	{0., 7.}	96.6	1.8891	newly 5 weeks cumul	143.	98.48	{7., 204.}	107.035	0.784129	newly 56 weeks cumul	211.	108.14	{7., 206.}	99.128	0.892667
45	newly 3 weeks spec	33.	8.28	{0., 12.}	96.1818	1.39815	newly 3 weeks cumul	81.	64.07	{6., 67.}	99.5185	0.59583	newly 5 weeks spec	24.	2.73	{0., 4.}	94.375	1.73309	newly 5 weeks cumul	141.	70.9	{7., 73.}	93.9291	0.765287	newly 54 weeks cumul	301.	74.86	{8., 73.}	89.6412	1.02725
46	newly 3 weeks spec	19.	9.9	{0., 20.}	81.0526	0.929098	newly 3 weeks cumul	57.	138.95	{16., 375.}	176.053	0.430983	newly 5 weeks spec	23.	2.44	{0., 5.}	94.5217	1.80932	newly 5 weeks cumul	104.	146.06	{16., 395.}	92.1538	0.369681	newly 61 weeks cumul	451.	148.75	{16., 419.}	72.592	0.749463
47	newly 3 weeks spec	5.	22.57	{0., 51.}	404.2	0.58195	newly 3 weeks cumul	19.	138.12	{13., 384.}	641.684	0.554294	newly 5 weeks spec	12.	6.67	{0., 23.}	106.25	1.28764	newly 5 weeks cumul	47.	156.81	{13., 519.}	273.553	0.406506	newly 59 weeks cumul	335.	166.87	{13., 539.}	95.0119	0.893401
48	newly 3 weeks spec	15.	18.42	{0., 50.}	162.267	1.12535	newly 3 weeks cumul	52.	131.89	{7., 342.}	230.788	0.485331	newly 5 weeks spec	38.	6.52	{0., 12.}	98.2632	2.02432	newly 5 weeks cumul	137.	150.04	{7., 418.}	136.307	0.73255	newly 54 weeks cumul	271.	160.84	{7., 501.}	106.192	0.935621
49	newly 3 weeks spec	12.	7.95	{0., 12.}	113.417	1.31544	newly 3 weeks cumul	36.	91.05	{7., 97.}	205.083	0.404379	newly 5 weeks spec	11.	2.64	{0., 5.}	100.909	1.63827	newly 5 weeks cumul	67.	98.02	{7., 105.}	134.507	0.494156	newly 58 weeks cumul	292.	102.61	{7., 114.}	94.5856	0.991486
50	newly 3 weeks spec	24.	6.29	{0., 11.}	93.625	1.55911	newly 3 weeks cumul	70.	99.24	{7., 413.}	139.857	0.581751	newly 5 weeks spec	19.	1.78	{0., 4.}	93.7895	1.7803	newly 5 weeks cumul	120.	104.51	{8., 413.}	108.708	0.70016	newly 55 weeks cumul	321.	107.39	{8., 413.}	83.5981	0.974
